# Bacterioplankton drawdown of coral mass-spawned organic matter

**DOI:** 10.1038/s41396-018-0197-7

**Published:** 2018-06-08

**Authors:** Ryan Guillemette, Ryo Kaneko, Jessica Blanton, Jasmine Tan, Matthias Witt, Samantha Hamilton, Eric E. Allen, Mónica Medina, Koji Hamasaki, Boris P. Koch, Farooq Azam

**Affiliations:** 1 Scripps Institution of Oceanography, UC San Diego, San Diego, CA USA; 2 National Institute of Polar Research (NIPR), Tokyo, Japan; 3 Bruker Daltonik GmbH, Bremen, Germany; 4 Smithsonian Tropical Research Institute, Bocas del Toro, Panama; 5 Pennsylvania State University, University Park, PA USA; 6 Atmosphere and Ocean Research Institute, The University of Tokyo, Tokyo, Japan; 7 Alfred Wegener Institute Helmholtz Centre for Polar and Marine Research, Bremerhaven, Germany

## Abstract

Coral reef ecosystems are highly sensitive to microbial activities that result from dissolved organic matter (DOM) enrichment of their surrounding seawater. However, the response to particulate organic matter (POM) enrichment is less studied. In a microcosm experiment, we tested the response of bacterioplankton to a pulse of POM from the mass-spawning of *Orbicella franksi* coral off the Caribbean coast of Panama. Particulate organic carbon (POC), a proxy measurement for POM, increased by 40-fold in seawater samples collected during spawning; 68% degraded within 66 h. The elevation of multiple hydrolases presumably solubilized the spawn-derived POM into DOM. A carbon budget constructed for the 275 µM of degraded POC showed negligible change to the concentration of dissolved organic carbon (DOC), indicating that the DOM was readily utilized. Fourier transform ion cyclotron resonance mass spectrometry shows that the DOM pool became enriched with heteroatom-containing molecules, a trend that suggests microbial alteration of organic matter. Our sensitivity analysis demonstrates that bacterial carbon demand could have accounted for a large proportion of the POC degradation. Further, using bromodeoxyuridine immunocapture in combination with 454 pyrosequencing of the 16S ribosomal RNA gene, we surmise that actively growing bacterial groups were the primary degraders. We conclude that coral gametes are highly labile to bacteria and that such large capacity for bacterial degradation and alteration of organic matter has implications for coral reef health and coastal marine biogeochemistry.

## Introduction

Marine organic matter can be conceptualized as a size continuum of biochemically diverse particles and molecules in labile, semi-labile, and refractory states [1–3]. Given its diverse and dynamic nature, organic matter has the ability to shape marine microbial communities [4, 5] including those associated with coral reefs [6, 7], which largely drive reef biogeochemical cycling [8–10] and can influence coral susceptibility to disease [11]. With the global health of coral reefs on a continued decline [12] it is imperative to better understand the microbial dynamics resulting from organic matter perturbation, which can directly influence these highly productive and bio-diverse marine habitats [13].

Studies of bacterial utilization of organic matter in coral reefs have primarily focused on dissolved organic matter (DOM), operationally defined as the organic matter within seawater filtrate from GF/F (glass fiber, 0.7 µm nominal-pore-size), 0.45 µm, or 0.2 µm filtration. Reports show that DOM composition, specifically of the mucus and sugars from coral and algal exudates, respectively, can affect bacterial specific growth rate, growth efficiency, and community composition [6, 7]. However, little is known about coral reef-associated microbial utilization of particulate organic matter (POM), operationally defined as the organic matter retained by GF/F filtration. Bacterial interaction with POM in the ocean is important for carbon cycling since POM can be transformed into DOM and subsequently respired by microbes [14]. Additionally, POM composition and concentration can influence microbial community dynamics [4, 5, 15], including the ability to select for virulence factors and pathogenic bacteria [16]. The few studies on POM degradation in coral reefs have suggested that it can be a highly labile nutrient source to microbes as indicated by dissolved oxygen depletion [17–19].

One particularly tractable, natural system for investigating microbial response to an influx of POM is coral mass-spawning. Through expulsion of gametes, vast amounts of organic matter are released that have been reported to produce slicks up to 5 km in length and 10 m in width [20]. On rare occasions, such events have caused severe hypoxia resulting in massive death for millions of fish and large areas of coral [21]. Conversely, less dramatic mass-spawning events are thought to provide nutrients to the generally oligotrophic reef waters [18].

A study off Heron Island, Great Barrier Reef in 2001 estimated that over 300 tons of particulate organic carbon (POC) was released during a mass-spawn event; the authors concluded that such episodic pulses of POM were important for fueling benthic microbial communities [19]. A series of studies conducted in 2005 found 3-fold to 11-fold increases in the concentration of POC in the water column, and increased oxygen consumption rates lasting for at least 1 week [18]. Primary production in the water column increased fourfold, likely a result of the observed increase in inorganic nutrients [22, 23]. Bacterial abundance in the field samples increased 2-fold [24], while laboratory experiments showed POC degradation rates of ~15% h^−1^ in seawater incubations that were enriched with filtered and killed gametes [18]. A study at Kaneohe Bay, Hawaii found a slight correlation of bacterial taxa to the presence of coral eggs [25]. While these studies have shown that coral gametes degrade in the sediment and water column, direct evidence that specifically links bacteria to gamete degradation has not been empirically demonstrated.

We designed a microcosm experiment to quantify the degradation of spawn-derived POM and investigated the bacterioplankton response to coral mass-spawning. Bacterial response was determined from bacterial production (BP) measurements based on incorporation rates of the thymidine analog, bromodeoxyuridine (BrdU), combined with 454 pyrosequencing of the BrdU-labeled 16S ribosomal RNA (rRNA) gene to estimate the bacterial carbon demand (BCD) of actively growing bacterial groups. Additionally, proxy measurements for POM degradation were determined by quantifying carbon flux through POC, colloidal organic carbon (colloidal carbon), and dissolved organic carbon (DOC) pools, along with the hydrolytic enzyme activities that we hypothesized would degrade the organic matter. Furthermore, via Fourier transform ion cyclotron resonance mass spectrometry (FT-ICR MS) we tested the hypothesis that the extracellular DOM (hereafter DOM) pool would contain a modified molecular composition as the organic matter moved between reservoirs.

## Materials and methods

### Study design

**Fig. 1:**
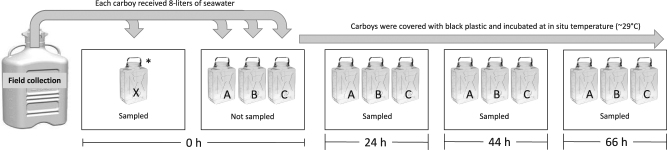
Microcosm sampling. Spawn and Non-spawn microcosm experiments were independently conducted in triplicate 10-liter polycarbonate carboys in the dark. Samples were taken at all time points for bacterial production, microbial abundance, and enzyme activities; the 0 and 66 h time points were also sampled for 16S rRNA sequencing, FT-ICR MS analysis, organic carbon and nitrogen concentration, and inorganic nutrient concentration. Carboys were well mixed by 5-times inversion before sampling with sterile-glass serological pipettes or a peristaltic pump. *One sampling was conducted at the 0 h time point; an 8-liter aliquot from the field sampling carboy was transferred into carboy “X” and then sampled to ensure that the seawater had been exposed to the same container composition and surface area

This study took place in 2014 at the Smithsonian Tropical Research Institute, Bocas del Toro, Panama, where each year coral of the genus *Orbicella* engage in well documented mass-spawn reproductive events [26]. Seawater was collected from within the 100×30 m transect of a long-term *Orbicella franksii* monitoring site (9° 19’ 38” N, 82° 12’ 14” W) to initiate three independent microcosm experiments, each conducted in triplicate. The field sampling was performed with an 80-liter carboy 3 days prior to spawning (September 8), during spawning while gametes were highly abundant in the water column (September 14), and 3 days after spawning (September 17). Microcosm experiments commenced within 8 h of sample collection and were sub-sampled at 0, 24, 44, and 66 h (Fig. 1). Additional field samples were taken for analysis 1 h before and 1 day after spawning (Table 1). All carboys were acid washed, MilliQ water-rinsed, and sample-rinsed prior to use.

**Table 1:** Concentrations of particulate, dissolved, and colloidal organic carbon; particulate organic nitrogen; and inorganic nutrients in field-collected seawater

Date (2014)	Field sample	POC^¶^ ( > 0.7 µm)	PON^¶^ ( > 0.7 µm)	POC:PON^†^ (ratio)	Colloidal carbon^†^ (0.2−0.7 µm)	DOC^¶^ ( < 0.2 µm)	TOC^†^ (µm)	Phosphate^¶^ (µm)	Silicate^¶^ (µm)	Nitrate^¶^ (µm)	Nitrite^¶^ (µm)	Ammonium^¶^ (µm)
**September 8**	**6 d pre-spawn**	12	2	5.5	BD	90	102	0.04	10.4	0.13	0.02	BD
September 14	1 h pre-spawn	11	2	5.3	BD	94	105	NS	NS	NS	NS	NS
**September 14**	**Spawn**	403 ± 7^a^	31 ± 2^a^	13.0	BD	112 ± 3^a^	515 ± 10	0.24	0.5	BD	0.04	BD
September 15	24 h post-spawn	6	2	2.8	BD	94	100	NS	NS	NS	NS	NS
**September 17**	**3 d post-spawn**	11	3	3.9	BD	96	107	0.4	0.4	BD	0.07	2.16

Values (in µM) were measured (¶) or calculated (†) from a single sample unless otherwise denoted

Sampling dates in bold indicate collections that were used to start a microcosm

*NS* not sampled, *BD* below detection

^a^SD calculated from two technical replicates of a single-biological replicate

Seawater samples from microcosms starting on September 8 and September 17 contained no gametes. These collections had ~40-fold less POC and ~15-fold less particulate organic nitrogen (PON) than the seawater collected during spawning on September 14. Additionally, these collections had comparable POC:PON ratios of 5.5 and 3.9, respectively, in comparison to 13.0 on the night of spawning (Table 1). Therefore, we combined data from these two microcosms and reported them together as “Non-spawn” microcosm throughout the manuscript. Seawater collected for the microcosm on September 14 did contain gametes and is referred to as “Spawn” microcosm.

### Particulate organic carbon and nitrogen analysis

Sample volumes of 500 ml were filtered onto pre-combusted 47 mm GF/F filters (Whatman, GE Life Sciences, Marlborough, MA) and frozen at −20 °C until processed (Supplementary Methods).

### Total, dissolved, and colloidal organic carbon

Seawater samples were each independently size fractionated through a pre-combusted GF/F filter and a 0.2 µm pore-size polytetrafluoroethylene filter (MilliporeSigma, Burlington, MA). A 40 ml aliquot from each fraction was separately collected into pre-combusted borosilicate vials and immediately acidified to pH 2 with hydrochloric acid (HCl). Each sample was quantified with a Shimadzu 500 V-CSN/TNM-1 analysis system (minimum limit of detection = 2.0 µM C) using a seven-point carbon standard with five sample injections (100 μl) per sample.

Organic carbon concentration in the 0.2 µm filtrate is reported as DOC. Colloidal carbon was calculated as the difference between organic carbon concentration in the GF/F (0.7 µm) filtrate and 0.2 µm filtrate. Total organic carbon (TOC) was calculated as the sum of POC, DOC, and colloidal carbon.

### Fourier transform ion cyclotron mass spectrometry

A peristaltic pump was used to filter each seawater sample (500 ml) through a GF/F filter then 0.2 µm pore-size polytetrafluoroethylene (PTFE) filter in sequence. The filters were each independently housed in a 47 mm filter-holder and were conjoined by polytetrafluoroethylene tubing. The 0.2 µm filtrate from each sample was independently collected into a polycarbonate bottle, acidified to pH 2 with HCl, then stored at −20 °C. DOM was extracted from the thawed 0.2 µm filtrate (320 ml per sample) using solid-phase extraction (SPE) cartridges (PPL, BondElut, 0.2 g). The cartridges were eluted with ~1 ml methanol. The exact volume of the extract was weighed and recalculated to volume (0.6–0.68 ml; equivalent to an enrichment factor of ~500). The DOC concentration in the extract [DOC_extract_] was determined with a Shimadzu analysis system as described above, and the carbon-based extraction efficiency (%) was calculated as 100 × [DOC_extract_]/enrichment factor × [DOC]. The average extraction efficiency for DOC was 47 ± 8%. FT-ICR MS analysis of the DOM was carried out as described previously [27] (Supplementary Methods).

### Bacterial production and loss

Triplicate samples were incubated with 20 nM BrdU, a thymidine analog. After incubation, BrdU incorporation was stopped by adding 100 µM thymidine, flash frozen, and stored at −80 °C until processed [28–30]. BrdU incorporation rates were converted to BP rates using an empirically derived conversion factor of 2 × 10^18^ bacterial cells produced per mole of incorporated-thymidine [31] and an applied linear regression of *y* = 0.69 × −0.81 for BrdU to thymidine incorporation rates [30]. Calculations for integrated BP rates, bacterial carbon production, and bacterial loss are detailed in the Supplementary Methods.

### Modeled apportionment of TOC drawdown

Refer to Supplementary Methods for terminology and equations used to calculate TOC drawdown; hypothetical bacterial growth efficiency (BGE); and estimation of BCD, bacterial respiration (BR), community respiration (CR), and non-bacterial respiration (NBR).

### Microbial cell enumeration

Seawater samples were fixed with 0.5% glutaraldehyde and stored at −80 °C until processed. Samples (1–2 ml) were filtered onto 0.2 μm pore-size polycarbonate filters and stained with DAPI Vectashield (Vector Labs., Burlingame, CA) for bacteria and sperm cell counts [32]. Samples (1 ml) were filtered onto 0.02 µm pore-size Anodisc filters (Whatman) then stained with SYBR Green I (Invitrogen, Carlsbad, CA) for virus-like-particle (VLP) counts [33]. Samples (5 ml) were filtered onto black 0.8 µm pore-size polycarbonate membranes and stained with DAPI Vectashield for protist enumeration. At least 200 cells from ten or more fields of view were counted by epifluorescence microscopy for bacteria and VLPs, while entire filters were examined for enumeration of protists.

### Bacterial cell size

DAPI fluorescence was used to measure the length and width of 100–600 individual bacterial cells per sample. An area cutoff of 0.1–2.0 µm^2^ was implemented to exclude VLPs and protists. Cell area was converted to biovolume (µm^3^ cell^−1^) based on the equation *V* = (*π*/4) × *W*^2^ × (*L* − *W*/3) [34]. Biovolumes were converted to protein content cell^−1^ using the power law function *P* = 88.6*^V0.59^ and multiplied by 0.86 [35] to calculate cell-specific bacterial carbon (cell-specific BC) (fgC cell^−1^) [36].

### Hydrolytic enzyme activities

Hydrolytic enzyme activities were assayed using fluorogenic substrates derived from 7-amino-4-methyl- coumarin (AMC) and 4-methyl-umbelliferone (MUF) [37, 38]. Protease activity was assayed as the hydrolysis rate of leucine-AMC. α-d-glucosidase, β-d-glucosidase, lipase, alkaline phosphatase, and chitinase activities were assayed as the hydrolysis rates of MUF labeled- α-d-glucoside, β-d-glucoside, oleate, phosphate and N-acetyl-β-d-glucosamine, respectively.

### Combined BrdU immunocapture and 454 pyrosequencing

Sample volumes (3 or 5-liter ) were taken for BrdU labeling and processing [39]. Incubation details and genomic DNA extraction methods are contained in the Supplementary Methods. BrdU-incorporated DNA represented the actively growing bacterial fraction (AGB) and was isolated from 1 µg of the Total DNA pool by immunocapture as previously described [39, 40]. DNA in both the Total and BrdU-labeled pools were processed and sequenced.

The hypervariable V1–V3 region of bacterial 16S rRNA gene sequences were amplified for 454 pyrosequencing with universal bacterial primers 27F (5’- AGAGTTTGATCMTGGCTCAG -3’) [41] and 519R (5’- GWATTACCGCGGCKGCTG -3’) [42]. Refer to the Supplementary Methods for detailed PCR amplification, pyrosequencing, data processing, and analysis methods. The average 454 pyrosequencing read length in our study was 388 base pairs. High confidence taxonomic assignments were made for 16S rRNA gene OTUs at family-level resolution. A small proportion of BrdU-labeled OTUs went undetected in the Total DNA pool, likely due to their relatively low abundance [43]. These OTUs are referred to as “active-but-rare” (ABR) [43].

Sequence data have been submitted to the NCBI Sequence Read Archive and are available under BioProject PRJNA388504. FASTA sequences for the reported AGB in Fig. 5c are available in Table S4.

### Inorganic nutrient analyses

Inorganic nutrient analyses (phosphate, silicic acid, nitrate + nitrite, nitrite, and ammonium) were performed on a Seal Analytical continuous-flow AutoAnalyzer 3 by the ODF Chemistry Laboratory (Scripps Institution of Oceanography, San Diego, CA).

### Statistical analysis

Statistics were conducted using GraphPad Prism version 7.0 (GraphPad Software, Inc.). A Shapiro–Wilk test of normality was implemented for each data set. Significant difference between treatments was evaluated with an unpaired *t*-test (two-tailed) for normally distributed data or a Mann–Whitney *U* test for data that was not normally distributed.

## Results

### Organic carbon concentration and POC:PON ratio

Seawater collected during spawning on September 14 was elevated approximately 5-fold in TOC in comparison to field samples collected during non-spawning conditions (Table 1). While POC constituted the predominant fraction of TOC in the September 14 spawning samples, insufficient replication precluded statistical evaluation for determining significant difference in POC concentration between field collections. However, the POC concentration during spawning (403 ± 7 µM) versus non-spawning conditions (6–12 µM) (Table 1) was substantial in comparison to a published compilation of global data that evaluated over 45 000 marine POC measurements (median POC = 7.36 µM) (Figure S1) [44]. Samples collected during spawning also had approximately threefold higher POC:PON ratio in comparison to samples from non-spawning conditions (Table 1). The calculated colloidal carbon concentration in all field-collected samples was below detection (Table 1).

**Fig. 2:**
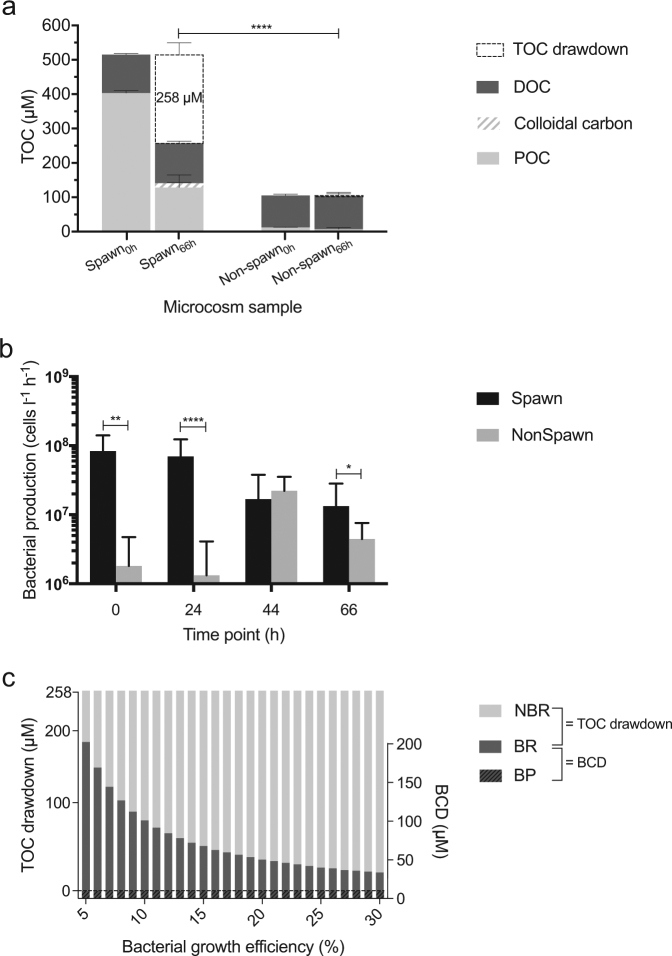
**a** Total organic carbon (TOC) concentration in microcosms Spawn and Non-spawn. TOC = particulate organic carbon (POC) + dissolved organic carbon (DOC) + colloidal carbon. For each fraction the error is represented by the mean ± SD of technical replicates (spawn_0h_, *n* = 2) or by single measurements of multiple biological replicates (Spawn_66h_, *n* = 3; Non-spawn_0h_, *n* = 2; Non-spawn_66h_, *n* = 6). Asterisks indicate significant difference in TOC drawdown (TOC_0h_–TOC_66h_) between treatments: ****p < 0.0001. **b** Bacterial production. Error bars represent ± SD of the mean (Spawn_0h_, *n* = 3; Spawn_66h_, *n* = 6; Non-spawn_0h_, *n* = 6; Non-spawn_66h_, *n* = 18). Asterisks indicate significant difference between treatments for each time point: *p < 0.05; **p < 0.01; ****p < 0.0001. **c** A range of bacterial carbon demand (BCD) (right *y*-axis) was estimated for microcosm Spawn on the basis of integrated BP rate measurements and a hypothetical range (5–30%) of bacterial growth efficiency. BCD = BP + bacterial respiration (BR). The model indicates that a broad range of BR and non-bacterial respiration (NBR) could have satisfied the observed TOC drawdown in microcosm Spawn (left *y*-axis)

The TOC concentration in microcosm Spawn_0h_ samples (515 ± 10 µM) was comprised of (403 ± 7 µM POC) + (112 ± 3 µM DOC) and colloidal carbon was below detection (Table 1, Fig. 2a). The observed TOC drawdown in microcosm Spawn (258 ± 42 µM) primarily resulted from a 68% reduction in POC, as colloidal carbon increased by 13 ± 2 µM, and the increase in DOC (4 ± 5 µM) was on the order of the error in our measurements (Fig. 2a). By comparison, microcosm Non-spawn TOC concentration underwent little change during incubation; the decrease in POC (5 ± 5 µM) and increase in DOC (3 ± 11 µM) remained within the error observed between replicates, and colloidal carbon remained undetectable (Fig. 2a). This resulted in a negligible drawdown of 2 ± 5 µM TOC in microcosm Non-spawn. The difference between TOC drawdown observed in microcosm Spawn and Non-spawn was significant (*p* < 0.0001) (Fig. 2a).

### Bacterial production and loss

Bacterial production rates were ~50-fold higher in microcosm Spawn_0h_ (*p* < 0.01), Spawn_24h_ (*p* < 0.0001), and Spawn_66h_ (*p* < 0.05) samples in comparison to Non-spawn (Fig. 2b). The BP rate in Non-spawn_44h_ samples exceeded those in Spawn_44h_, but the difference was not statistically significant. Integration of BP rates shows that microcosm Spawn produced approximately 6-fold more bacterial cells and biomass in comparison to Non-spawn (*p* < 0.0001), and that a large proportion of bacterial cells were lost during incubation (Spawn, 50 ± 18%; Non-spawn, 42 ± 16%) (Table 2). Cell-specific BC measured at each respective time point showed no statistical difference between treatments (Table 2).

**Table 2:** Cell-specific bacterial carbon, integrated bacterial production and bacterial loss in microcosm samples

		Cell-specific BC	Bacterial production^¶^		Bacterial loss^¶^		
Microcosm	Time (h)	(fgC cell^−1^)	(10^9^ cells × l^−1^)	(µM C)	(10^9^ cells × l^−1^)	(µM C)	(%)
S	0	42.7 ± 18.3^†^	**3.0** **±** **0.62**	**9.8** **±** **2.1**	**2.2** **±** **0.99**	**7.0** **±** **3.3**	50 ± 18
	24	31.8 ± 1.32					
	44	38.3 ± 4.06					
	66	47.8 ± 2.22					
NS	0	40.1 ± 0.94	0.57 ± 0.30	1.6 ± 0.86	0.70 ± 0.47	1.8 ± 1.3	42 ± 16
	24	28.8 ± 1.10					
	44	30.2 ± 2.31					
	66	36.6 ± 3.32					

Cell-specific bacterial carbon (cell-specific BC) is represented by the mean ± SE of 406 to 2408 single-cell measurements per biological replicate unless otherwise denoted (Spawn_0h_, *n* = 1; Spawn_66h_, *n* = 3; Non-spawn_0h_, *n* = 2; Non-spawn_66h_, *n* = 6)

Insufficient replication precluded statistical evaluation between treatments for cell-specific BC at the 0 h time point

Bacterial production and bacterial loss are reported as the mean ± SD (spawn, *n* = 3; Non-spawn, *n* = 6)

Values in bold denote significant difference between treatments: bacterial production (*p* < 0.0001); bacterial loss (*p* < 0.01)

^†^SD calculated from single-cell measurements in one biological replicate

^¶^Integrated rates (66 h)

### Modeled apportionment of the observed TOC drawdown

Our sensitivity analysis shows an estimated range of BCD (33–196 µM) for microcosm Spawn (Fig. 2c). Under the assumptions used, this model demonstrates that the TOC drawdown in microcosm Spawn could have been satisfied by a broad range of combined BR (23–186 µM) and NBR (71–234 µM) (Fig. 2c) (further discussed below).

### Microbial abundance

**Fig. 3:**
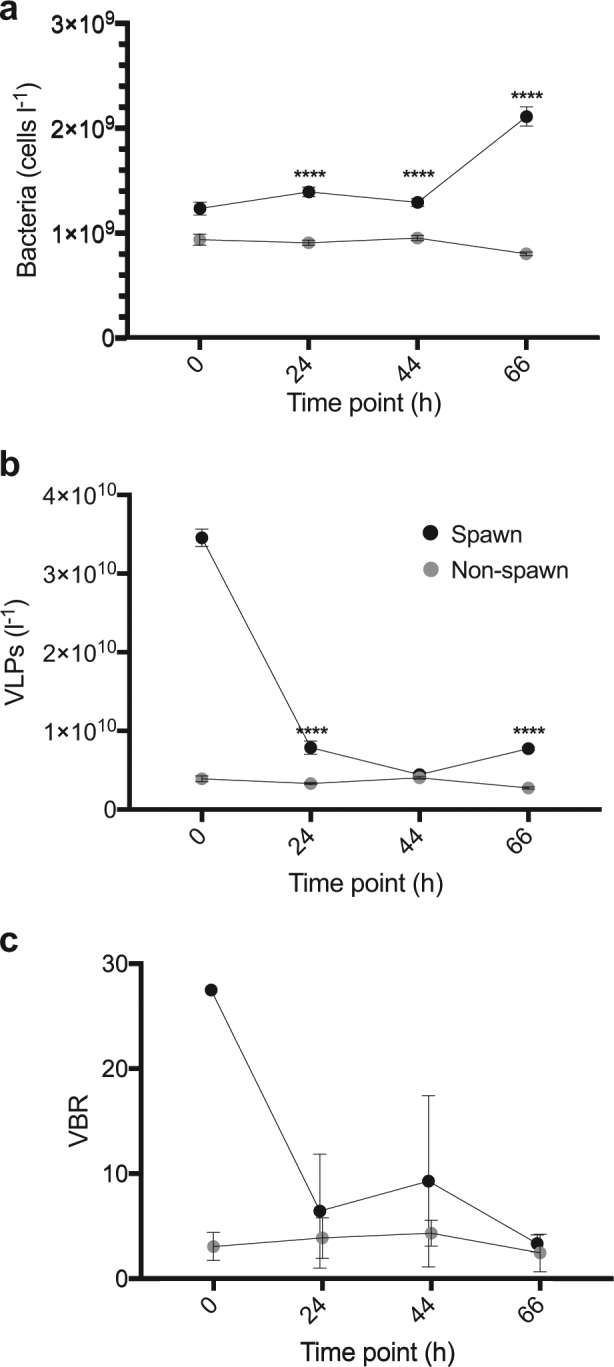
Bacterial abundance (**a**) and virus-like-particle (VLP) abundance (**b**) in microcosm Spawn (black) and Non-spawn (gray) samples. At least 20 fields of view were enumerated for each microscopy sample (Spawn_0h_, *n* = 1; Spawn_66h_, *n* = 3; Non-spawn_0h_, *n* = 2; Non-spawn_0h_, *n* = 6). Error bars represent the standard error of the mean. **c** Virus-to-bacteria ratio (VBR). Error is represented by ± SD of the mean. Asterisks indicate significant difference between treatments for each time point: ****p < 0.0001. Insufficient replication precluded statistical evaluation between treatments at the 0 h time point for data in (**a**–**c**)

Bacterial abundance was 1.3-fold to 2.6-fold higher (*p* < 0.0001) in microcosm Spawn_24h_, Spawn_44h_, and Spawn_66h_ samples in comparison to Non-spawn samples at the same time points (Fig. 3a). VLP abundance in microcosm Spawn was elevated at each sampling time point in comparison to Non-spawn, however only the Spawn_24h_ and Spawn_66h_ samples were significantly higher (*p* < 0.0001) (Fig. 3b). Interestingly, the VLP abundance at Spawn_0h_ decreased 3-fold within 24 h (Fig. 3b) suggesting that the VLPs were consumed or degraded. We also note that the virus-to-bacteria ratio (VBR) was higher at all time points for microcosm Spawn samples in comparison to Non-spawn, however there was no significant difference between treatments (Fig. 3c). Protist abundance was approximately 2-fold higher (*p* < 0.01) in microcosm Spawn samples (Spawn_0h_, 3.6 ± 0.6 × 10^6^ cells l^−1^; Spawn_66h_, 2.2 ± 0.3 × 10^6^ cells l^−1^) in comparison to microcosm Non-spawn samples at the same time point (Non-spawn_0h_, 1.8 ± 0.7 × 10^6^ cells l^−1^; Non-spawn_66h_ 1.1 ± 0.5 × 10^6^ cells l^−1^). At Spawn_0h_, the sperm cell abundance was 3.9 ± 0.9 × 10^7^ cells l^−1^. No sperm cells were detected in the subsequent microcosm Spawn samples or in any of the Non-spawn samples.

### Hydrolytic enzyme activities

**Fig. 4:**
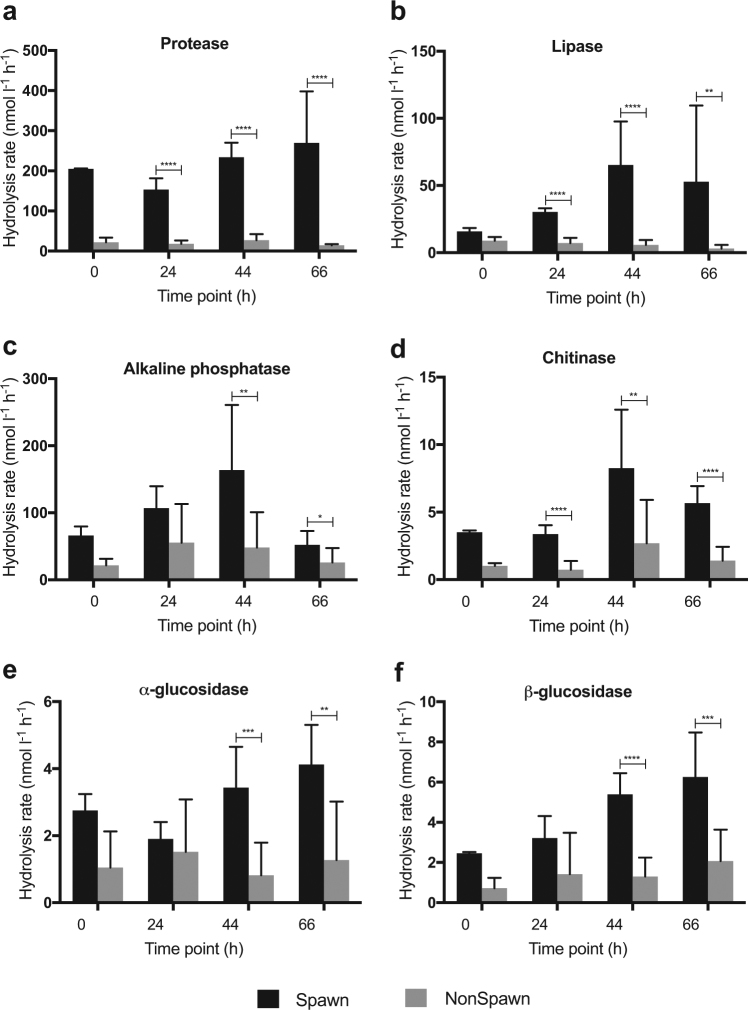
Hydrolysis rates of enzyme substrates for microcosm Spawn samples (black) and Non-spawn samples (gray). **a** Protease; **b** Lipase; **c** Alkaline phosphatase; **d** Chitinase; **e** α-glucosidase; and **f** β-glucosidase. Error bars represent ± SD of the mean (Spawn_0h_, *n* = 2; Spawn_24,44,66h_, *n* = 6; Non-spawn_0h_, *n* = 4; Non-spawn_24,44,66h_, *n* = 12). Asterisks indicate significant difference between treatments for each time point: *p < 0.05; **p < 0.01; ***p < 0.001; ****p < 0.0001. Insufficient replication precluded statistical evaluation between treatments for the 0 h time point

Hydrolytic enzyme activities in microcosm Spawn samples were greater than Non-spawn at each time point for all measured enzymes (Fig. 4). Protease activity was particularly elevated (up to 19-fold) (*p* < 0.0001) (Fig. 4a) and lipase activity was 1.5-fold to 11-fold higher (*p* < 0.01) in Spawn versus Non-spawn samples (Fig. 4b).

### Actively growing bacterial community analysis

Microcosm Spawn and Non-spawn samples contained a large number of unique taxa relative to their specific treatment, however in terms of mean relative abundance the vast majority of sequences in the 0 h samples (>97%) (Figure S2 a) and 66 h samples (>91%) (Figure S2 b) were assigned to taxa found in both treatments.

**Fig. 5:**
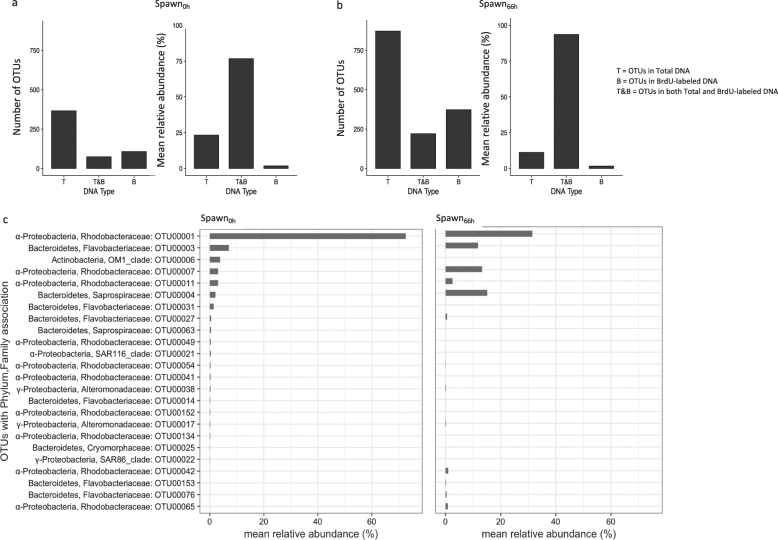
Actively growing bacterial community analysis. Spawn_0h_ (**a**) and Spawn_66h_ (**b**) show the number of taxa and summed mean relative abundances of OTUs. T Total DNA, B BrdU-labeled DNA, T&B total & BrdU-labeled DNA. **c** Mean relative abundance of BrdU-labeled taxa in microcosm Spawn samples (Spawn_0h_, *n* = 1; Spawn_66h_, *n* = 3). BrdU-labeled OTUs that have ≥0.1% relative abundance are shown, and taxa from Spawn_66h_ samples had to be found in at least two of the three replicates. Class designations: α, Alphaproteobacteria; γ, Gammaproteobacteria

Over 75% of the summed mean relative abundances from the sequenced Total DNA and BrdU-labeled DNA pools of microcosm Spawn samples were assigned to common OTUs (Fig. 5a,b). Taxa that were not BrdU-labeled, but were present in the Total DNA pool comprised much of the remaining mean relative abundance for Spawn_0h_ samples (23.3%) (Fig. 5a) and Spawn_66h_ samples (17.8%) (Fig. 5b). We also found OTUs in the BrdU-labeled DNA pools that went undetected in the Total DNA. These ABR bacterial groups had mean relative abundances of ~2% in the Spawn_0h_ samples and ~2% in the Spawn_66h_ samples (Figure S3).

Eight OTUs were the most abundant AGB in microcosm Spawn samples, which included members of the families *Rhodobacteracea*, *Saprospiraceae*, *Flavobacteriaceae*, and *Alteromonadaceae* (Fig. 5c). The AGB in Spawn_66h_ samples included five OTUs that arose from below detection at Spawn_0h_, including additional members of the *Rhodobacteracea*, *Flavobacteriaceae*, and *Alteromonadaceae*. Eleven OTUs that were present as AGB at Spawn_0h_ fell below detection in the Spawn_66h_ samples, including OTUs of the *Rhodobacteracea*, *Saprospiraceae*, *Flavobacteriaceae*; as well as the only members of the *OM1*, *SAR116*, *Cryomorphaceae*, and *SAR86 clades*. The abundance of AGB represented by OTUs in the Spawn_66h_ samples shifted, as total *Rhodobacteracea* dropped from 80.0 to 49.1%, while *Saprospiraceae* increased from 2.6 to 12.2%, and *Flavobacteriaceae* increased from 8.5 to 11.8%. The fourth most abundant OTU, an *Alteromonadacea*, increased slightly from 0.27 to 0.33%. These four dominant families also represented the highly abundant AGB that were particle-associated in the microcosm Spawn_66h_ samples (Figure S4). Further taxonomic classification of differentially abundant OTUs at the genus or species level did not resolve with high confidence suggesting novel groups of marine bacteria, specifically Alphaproteobacteria (*Rhodobacteraceae*) and Bacteroidetes (*Flavobacteriaceae* and *Saprospiraceae*), were associated with Spawn samples.

### FT-ICR MS analysis of dissolved organic matter

**Fig. 6:**
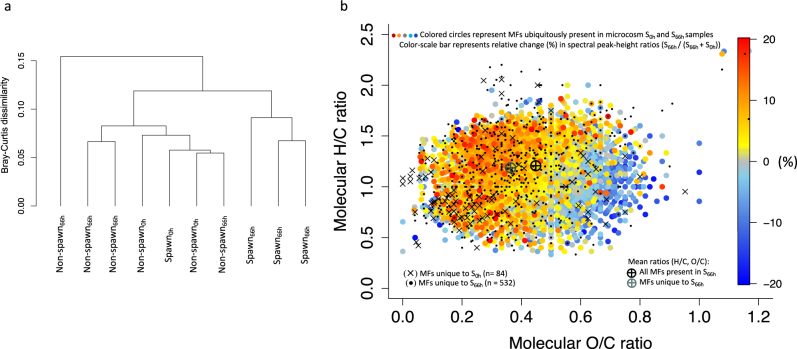
FT-ICR MS analysis of dissolved organic matter (see Table S1 for sample details; Table S2 for data summary). **a** Cluster dendrogram based on the relative spectral peak-heights of all molecular formulas (MFs) identified in microcosm samples. Distance calculated by Bray–Curtis measure and the complete-linkage method. **b** Van Krevelen diagram showing identified MFs in microcosm Spawn samples (Spawn_0h_, *n* = 1; Spawn_66h_, *n* = 3). Black crosses (*n* = 82), MFs unique to microcosm Spawn_0h_; black dots (*n* = 532), MFs unique to Spawn_66h_. Colored dots (*n* = 3 007), MFs ubiquitously identified in both Spawn_0h_ and Spawn_66h_ samples (all replicates). Color scale represents relative change from Spawn_0h_ to Spawn_66h_, derived by calculating relative peak-height ratios for each molecular formula (Spawn_66h_/(Spawn_66h_ + Spawn_0h_)). Ratios as “percent relative change” are shown: red, 20% relative increase; gray, no change; blue, 20% relative decrease. Black crosshair, weighted-mean element ratio of all MFs detected in microcosm Spawn_66h_ samples (*n* = 16,907); gray crosshair, weighted-mean element ratio of the unique MFs (*n* = 532) in the same samples. S, microcosm Spawn.

Cluster analysis of the relative spectral peak-heights for all molecular formulas identified in the microcosms (Table S1) shows that Spawn_66h_ samples clustered together, and distinct from, Spawn_0h_ and all Non-spawn samples (Fig. 6a). The most marked differences between identified molecular formulas in Spawn_0h_ and Spawn_66h_ samples was represented by the formulas unique to Spawn_66h_ (*n* = 532). Most of these unique formulas (*n* = 404) contained nitrogen or sulfur atoms (heteroatoms) (Table S2). Additionally, the weighted-means of the elemental ratios (O/C, H/C) for these unique formulas shows a relative decrease in oxygen content, and a slight decrease in saturation, when compared to the weighted-mean for all of the molecular formulas present in Spawn_66h_ samples (Fig. 6b).

The color density scale (±20%) (Fig. 6b) and the weighted-means of the elemental ratio (O/C) (Table S2) shows that slightly less oxidized formulas remained after 66 h incubation for the molecular formulas ubiquitously identified in all microcosm Spawn samples (at 0 and 66 h). There were also five molecular formulas ubiquitously present in all microcosm Spawn samples that were not present in any of the microcosm Non-spawn samples (Table S3), which suggests that there were unique formulas to the mass-spawning event.

## Discussion

### Increased POC and PON concentration during mass-spawning

We hypothesize that coral gametes, primarily the eggs, were responsible for the ~40-fold increase in POC and ~15-fold increase in PON of seawater samples collected during spawning (Table 1). When spawning commenced, clouds of released gametes were observed by SCUBA and the samples contained eggs that were visibly abundant. Furthermore, the POC:PON ratio in microcosm Spawn was approximately 3-fold higher than Non-spawn samples (Tables 1) and approximately 2-fold higher than the median POC:PON ratio (6.6) of over 45,000 global samples [44]. Previous studies have reported that lipid-rich coral eggs [45] elevated the POC:PON ratio of post-spawn seawater to levels comparable to those in our study [18] (Table 1), further supporting our hypothesis.

It is noteworthy that seawater samples collected 1 d and 3 d post-spawning had POC and DOC concentrations that were similar to pre-spawning samples (Table 1). The current at our study site may have displaced spawn-derived organic matter from the reef proper, particularly the gametes and developing larvae, which are buoyant and can remain in the surface-water for days [19, 20, 45]. Advection implies that metabolic signatures of the mass-spawning event were not confined exclusively to the sampling location and that any impacts on the carbon cycle may also have influenced water masses away from our site. Future studies might consider sampling away from the reef to assess the broader impacts that spawning may have on the surrounding coastal marine ecosystem.

### POC degradation and TOC drawdown in microcosm Spawn

The observed degradation of 275 ± 36 µM POC in microcosm Spawn (Fig. 2a) may have resulted from enzymatic hydrolysis of gametes. We found significantly elevated lipase and protease activity (Fig. 4a, b), and further note that coral eggs and sperm are largely comprised of lipid and protein [45]. While the source of enzymes was not determined, it is well known that marine bacteria are capable of high enzymatic hydrolysis rates [37, 38], and to our knowledge no enzymes have been found directly associated with coral gametes.

The majority of degraded POC was presumably respired by bacteria and non-bacteria (e.g., zooplankton and protists) resulting in the observed TOC drawdown (Fig. 2a). As shown in Fig. 2a, the increase in DOC was on the order of the error in our measurements and can be considered negligible. Additionally, only a small quantity of carbon appeared in the colloidal fraction (Fig. 2a). This suggests that consumers of the degraded POM readily utilized it through tight-coupling mechanisms [46, 47].

Our model shows that BCD (primarily BR) could theoretically account for a large proportion of the TOC drawdown (Fig. 2c). To be conservative, we used a range of BGE (5–30%) that is commonly reported in marine studies [14, 48] to estimate BR. However, the literature shows that BR generally constitutes 40–80% of CR in seawater (median = 45%) [48]. Based on our model, an overall BGE of <8% would have been required for BR to have constituted at least 40% of CR (Fig. 2c). Therefore, it is possible that BGE of >8% in our model underestimates the contribution of BR.

The remaining proportion of degraded POC appears to have accumulated in the colloidal carbon pool, which may be due to a number of factors. One possibility is that a portion of the spawn-derived POM transitioned through an intermediate colloidal phase during degradation. Additionally, bacterial lysate that may have accumulated from the 7.0 ± 3.3 µM C of bacterial loss (Table 2) could have constituted a substantial proportion of the observed increase in colloidal carbon (Fig. 2a). It is also possible that a portion of the DOM underwent spontaneous assembly into colloids [49, 50].

Colloids have been reported at concentrations of up to 10^8^ ml^−1^ in seawater samples [51, 52] and may comprise ~10% of the total DOM pool [53]. Organic matter within the colloidal size range could be directly consumed by protists or further degraded and taken up by bacteria. It has been suggested that colloidal particles are a significant source of nutrient to bacteria [54]. Future studies might consider colloids when quantifying carbon flux, as marine bacteria likely use different mechanisms for processing truly dissolved versus colloidal organic matter—both contained in operationally defined DOM samples of GF/F filtrate.

### Actively growing bacteria as the primary degraders

Given that BrdU was used as the tracer for BP rate measurements and the AGB community analysis, the AGB found in microcosm Spawn samples (Fig. 5c) were presumably responsible for the greatest proportion of our estimated BCD (Fig. 2c). AGB community analysis revealed Bacteriodetes and Proteobacteria (class Alphaproteobacteria) as the dominant phyla in microcosm Spawn (Fig. 5c). Both phlya have been reported as actively growing via BrdU incorporation in a previous study [55], and are known responders to organic matter enrichment by coral- and algal-reef-associated bacteria [6, 11]. The dominant AGB in microcosm Spawn_0h_ and Spawn_66h_ samples were comprised of *Rhodobacteraceae*, *Flavobacteriaceae*, and *Saprospiraceae* families, and were specifically represented by OTUs 001, 003, 004, 007, and 011 (Fig. 5c). These OTUs were also identified in both the filtrate-associated and particle-associated fraction of Spawn_66h_ samples (Figure S4). It is possible that the particle-associated taxa may have released progeny into the surrounding water to feed on energy-rich plumes of DOM generated by degraded POM [56–58], or that the existing, free-living bacteria also fed on such hydrolysate plumes [59]. It has been suggested that such strategies could account for up to 50% of global ocean BCD [59].

The overwhelmingly abundant OTU in microcosm Spawn, OTU 001 (*Rhodobacteraceae*), constituted 72.5 and 31.5% of the relative abundances for AGB in Spawn_0h_ and Spawn_66h_ samples, respectively (Fig. 5c). This sustained dominance, particularly at 0 h when BP was the highest (Fig. 2b), suggests that OTU 001 may have been responsible for the greatest proportion of BCD. As the AGB community composition shifted from microcosm Spawn 0 to 66 h (Fig. 5c) there was a concomitant reduction in BP (Fig. 2b). At 66 h, the relative abundance of OTU 001 dropped by over 50%, and OTUs 003 (*Flavobacteriaceae*), 007 (*Rhodobacteraceae*), and 004 (*Saprospiraceae*) increased by 2-fold to 5-fold, while BP dropped by approximately 4-fold . This may have led to a shift in the BGE of specific taxa, and/or in the overall BGE of the bacterial community, but these possibilities were not investigated. We acknowledge that the use of universal primers does not necessarily mean that all taxa contained in microcosm Spawn were detected, therefore no attempt was made to quantitatively apportion BP or BCD to specific taxa.

### Active-but-rare bacterial taxa in microcosm Spawn

Active-but-rare bacterial taxa have been previously described as OTUs that are either found at the tail-end of the rank-abundance curve for the sequenced Total DNA pool, but make-up a large proportion of the OTUs in the BrdU-labeled DNA pool; or they emerge in samples where bacteria are rapidly dividing, but their abundances are kept in check by tight predatory controls [43, 60]. The observation of significantly elevated BP rates during microcosm Spawn (Fig. 2b), coupled with our finding that ~50% of bacteria were lost during incubation (Table 2), seems to support the latter scenario for the ABR taxa found in our study (Figure S3). Although the ABR OTUs never reached high relative abundance, they could have made significant contributions to carbon flux as their rapidly assimilated cell carbon could have been lysed or consumed by grazers and cycled into the microbial loop.

Protist grazing, viral lysis, and bacterial antagonism are considered to be the primary predatory controls that regulate bacterial abundance [61]. The VBR of 28 in the microcosm Spawn_0h_ sample was approximately three fold higher than all other Spawn samples, but insufficient replication precluded statistical evaluation for determining significant differences between treatments (Fig. 3c). However, we note that this value is approximately 4-fold higher than the mean VBR reported in a study of 223 global coral reef samples (range, 2–25; mean, 7.4) [62]. The high VBR at Spawn_0h_ may suggest that bacteria were subject to viral infection early in the microcosms when BP rates were the highest. We also found highly abundant families of *Saprospiraceae* in the microcosm Spawn samples (Fig. 5c), which are known to prey on other bacteria using a method known as ‘ixotrophy’ [63]. Additionally, the protists that were present in microcosm Spawn likely contributed to the regulation of bacterial abundance. Protist communities are reported to ingest ~5 × 10^7^ bacteria l^−1^ h^−1^ [64].

### Alteration of DOM in microcosm Spawn revealed by FT-ICR MS analysis

Ultrahigh resolution FT-ICR MS analyses have been applied to characterize the molecular composition of complex DOM samples [65, 66] including the alteration of DOM pools by microbial activity [67, 68]. We predicted that the DOM composition in our microcosm Spawn samples would differ after incubation due to bacterial degradation of gametes. The molecular formulas unique to microcosm Spawn_66h_ samples (Fig. 6b) were dominated by heteroatom-containing (sulfur and nitrogen) formulas (Table S2), which are likely to be metabolized faster than the refractory DOM background [69]. Our finding is similar to the mesocosm results of a previous DOM study where nitrogen and sulfur heteroatom-containing compounds were generated via microbial alteration of organic matter [68]. Metabolic processes associated with the AGB in our study, along with the elevation of multiple hydrolases likely contributed to the observed alteration of the DOM pool.

Lastly, we note that our analysis which identified the five molecular formulas unique to microcosm Spawn samples (Table S3) did not consider the entirety of molecules present, as there are biases inherent to DOM size fractionation, electrospray ionization, and DOM extraction methods [70]. However, we suggest that given the conservative criteria used (formulas had to be found in all replicates of all Spawn samples and not present in any of the Non-spawn samples), these molecular formulas may be a good starting point for future research related to: (1) signaling molecules for coral to synchronously release mass-spawn; (2) molecular settling cues for developing larvae; or (3) molecular indicators that corals have spawned.

## Conclusion

Our microcosms study showed that the microbial community in the water overlying coral reef could readily respond to spawn input even though this input was mainly comprised of particulate phase. While the underlying mechanisms were not fully examined here, the elevation of multiple hydrolases was presumably responsible for the transition of the particulate phase into DOM to meet the large estimated BCD and an impressive magnitude of carbon flux into the microbial loop. Our study of the AGB community dynamics via BrdU incorporation led to the finding that *Rhodobacteraceae*, *Flavobacteriaceae*, and *Saprospiraceae* taxa were capable of utilizing the episodic organic matter input. Further, we suggest these AGB played a major role in the degradation of POM and likely contributed to the alteration of DOM as identified by high resolution mass spectrometry (FT-ICR MS) analysis. These findings of tight coupling between bacteria and organic matter are highly relevant to coral reef health and to the broader biogeochemical dynamics of coastal marine systems subject to large episodic input of organic matter.

## Electronic supplementary material

Supplementary Methods

Figure S1

Figure S2

Figure S3

Figure S4

Table S1

Table S2

Table S3

Table S4

Supplementary Figure Legends
