# Supplementary material for: Bacterioplankton drawdown of coral mass-spawned organic matter
Source: ISME J. 2018 Jun 8;12(9):2238–51. doi: 10.1038/s41396-018-0197-7 (PMC6092384; doi:10.1038/s41396-018-0197-7)
Supplement: Supplementary file 1 — Supplementary Methods [file 41396_2018_197_MOESM1_ESM.docx]

**Supplementary Methods**

This supplementary information contains expanded Materials and Methods.

*Particulate organic carbon and nitrogen analysis*

The thawed GF/F filters were placed in a desiccator in the presence of concentrated HCl vapor (12 M) to remove inorganic carbonates and then dried at 50 °C. Carbon and nitrogen was analyzed by high-temperature combustion using an organic elemental analyzer (model CEC 440HA; Control Equipment Corp.) by the Marine Science Institute Analytical Laboratory (University of California, Santa Barbara, CA). Duplicate filters with no sample were handled, stored, and processed in tandem and used as procedural blanks. Duplicate filters receiving MilliQ water were used as operational blanks. Blank values were subtracted from the field and experimental sample measurements. The limit of detection for carbon and nitrogen ranged from 0.1 to 0.4 µM with precision of ± 0.3 wt% and an accuracy of ± 0.3 wt%.

*Bacterial production*

Bacterial production (BP) rates at the 0, 24, 44, and 66 h time points were integrated to estimate the total number of bacteria produced per unit volume over the microcosm duration, BP_Total_.

${BP}_{Total}=\int_{t=0}^{t=66} BP(t)dt=\sum_{n=1}^{n=3} \left( \frac{(BP\left( t_{n} \right)+BP(t_{n+1})}{2} \right)*\left( t_{n+1}-t_{n} \right)$ (1)

Terms: BP_Total_ = cells l^-1^; BP = cells l^-1^ h^-1^; t_1,2,3,4_ = 0, 24, 44, and 66 h time points, respectively.

BP_Total_ (Eq. 1) was then multiplied by cell-specific bacterial carbon (cell-specific BC) to determine the total production of bacterial carbon, BP_Total_Carbon_.

*BP_Total_Carbon_ = BP_Total_ * Cell-specific BC*  (2)

Terms: BP_Total_Carbon_ = µM C; BP_Total_ = cells l^-1^; Cell-specific BC = fgC cell^-1^

*Calculation of bacterial carbon demand and bacterial respiration*

We aimed to estimate the proportion of organic carbon that was utilized in the microcosms to support bacterial carbon demand (BCD); carbon consumed for bacterial production (BP) and bacterial respiration (BR).

*BCD = BP + BR* (3)

Given the methodological limitations of measuring BR in natural assemblage (Robinson, 2008), we used a sensitivity analysis to estimate BCD based on our calculated BP (Eq. 2) and a hypothetical range of bacterial growth efficiency (BGE). BGE is defined as the proportion of BCD that is used for BP.

*BGE = BP / BCD* (4)

The range of BGE (5–30%) was selected as input into our model based on previously reported BGE values for coastal seawater bacteria (Del Giorgio & Cole, 1998). This type of analysis has been applied in several studies (Meyer et al, 1987, Kirchman et al, 1991, Smith et al, 1995, Del Giorgio & Cole, 1998).

*BCD_Max_ = BP / .05* (5)

*BCD_Min_ = BP / .30* (6)

We then used BCD_Max_ (Eq. 5) and BCD_Min_ (Eq. 6) to estimate the maximum and minimum range of BR.

*BR_Max_ = (BP / .05) - BP* (7)

*BR_Min_ = (BP / .30) - BP* (8)

*Modeled apportionment of the observed total organic carbon (TOC) drawdown to BR and non-bacterial respiration (NBR)*

The difference between TOC concentration at 0 h and 66 h was reported as TOC drawdown for each microcosm.

*TOC drawdown = (TOC_0h_ - TOC_66h_)* (9)

Since all of the organic carbon (including biomass) in the microcosm samples was accounted for in the TOC, then the respiration of the entire heterotrophic community, community respiration (CR), was effectively responsible for the observed TOC drawdown. Community respiration can also be conceptualized as the sum of BR and NBR. Therefore, we hypothesized that the observed TOC drawdown in our study resulted from combined BR and NBR. We used our estimated BR_Max_ (Eq. 7) and BR_Min_ (Eq. 8) to estimate NBR_Max_ and NBR_Min_.

*NBR_Min_ = TOC drawdown - BR_Max_* (10)

*NBR_Max_ = TOC drawdown - BR_Min_* (11)

Thus, our modeled apportionment of the observed TOC drawdown was partitioned to a broad range of combined BR (Eq. 7, 8) and NBR (Eq. 10, 11) estimates.

*Bacterial loss*

Bacterial loss was calculated as the difference between bacterial abundance at the 66 h time point (BA_66h_) and the sum of BP_Total_ (Eq. 1) plus bacterial abundance at the 0 h time point (BA_0h_) (Smith et al, 1995).

*Bacterial loss = (BP_Total_ + BA_0h_) - BA_66h_*  (12)

Terms: Bacterial loss, BP_Total_, BA_0h_, BA_66h_ = cells l^-1^

Bacterial loss (Eq. 12) was multiplied by the mean cell-specific BC to estimate bacterial carbon loss during the 66 h incubation.

*Bacterial carbon loss = Bacterial loss * Cell-specific BC*  (13)

Terms: Bacterial carbon loss = µM C; Bacterial loss = cells l^-1^; Cell-specific BC = fgC cell^-1^

*Fourier transform ion cyclotron mass spectrometry (FT-ICR MS)*

Prior to analysis the DOM extracts were diluted with methanol:water (1:1, v:v) to reach a final concentration of 2 µmol DOC_extract_ / mL MeOH. Samples were ionized by electrospray ionization (ESI, Apollo II electrospray ionization source, Bruker Daltonik, Bremen, Germany) in negative mode at an infusion flow rate of 120 μl h^−1^ with a Fourier transform ion cyclotron resonance mass spectrometer (SolariX, Bruker Daltonik, Bremen, Germany) equipped with a 12 T refrigerated, actively shielded, superconducting magnet (Bruker Biospin, Wissembourg, France). Two hundred scans were added to one mass spectrum. The magnitude threshold for the peak detection was set to a signal-to-noise ratio of > 4. Mass spectra were recalibrated internally with compounds that have been repeatedly identified in marine DOM samples (Koch et al., 2008; Flerus et al., 2011; m/z: 339.10854, 369.15549, 411.12967, 469.13515, 541.15628, 595.23962, 611.19814, 651.22944). The average mass accuracy of the internal calibrants was below 0.1 ppm.

*FT-ICR MS data evaluation*

All ions were singly charged as confirmed by the spacing of the related ^12^C_n_ and ^13^C^12^C_n−1_ mass peaks. The spectra were evaluated in the mass range of 200–650 m/z. The base peak (either 407.1348 or 411.1296 m/z for all samples) was defined as 100% and relative intensities for all other peaks were calculated accordingly. Molecular formulas were calculated from m/z values allowing for elemental combinations ^12^C_0-∞_ ^13^C_0−1_ ^1^H_0-∞_ ^14^N_0−6_ ^15^N_0−1_ ^16^O_0-∞_ ^32^S_0−3_ ^34^S_0−1_ ^31^P_0-3_ and a mass accuracy threshold of |Δm| ≤ 0.2 ppm. The double bond equivalent (DBE) of a valid neutral formula had to be an integer value and the “nitrogen rule” was applied (Koch et al, 2007). Formulas which were either detected in two process blanks (PPL extraction of ultrapure water) or contained in the list of potential surfactants (Lechtenfeld et al, 2013) were removed from the entire data set. Formulas containing a ^13^C, ^15^N or ^34^S isotopes that did not correspond to a parent formula (^12^C, ^14^N, ^32^S) were also removed from the data set. Two samples in the original analysis (from microcosm Non-spawn_66h_) were excluded due to a disproportionately lower number of spectral peaks in comparison to all other samples, a trend that suggested contamination by salt remnants.

As an additional level of formula validation, all formulas were sorted according to DBE and ppm. A small proportion of formulas corresponded to very high DBE values, many of which were false assignments of sulfur containing compounds (as identified by the peak ratio of the parent and daughter ions). We therefore used DBE ≤ 30 as an additional cut-off which resulted in an unambiguous assignment for the complete data set. The distribution of mass accuracy also showed that the majority of the assigned formulas were well within the 0.2 ppm threshold. After these validation steps, we excluded the stable isotopes ^13^C, ^15^N, and ^34^S because they only represented duplicates of the parent formulas for subsequent sample comparisons. Intensity weighted average (wa) molecular masses and element ratios were calculated from the base-peak normalized peak-heights. For formulas with a very high relative intensity, the isotope ratio provided an additional level of formula validation (Koch et al, 2007). It should also be considered that the solid-phase extraction method applied in this study does not cover the entirety of chemical compounds in the samples. Therefore, a focus of our evaluation procedure was on formulas that ubiquitously occurred in the control and treatment samples, and their relative peak-height changes. This excludes the possibility that differences between samples were caused by a shift of the analytical window.

*Combined BrdU immunocapture and 454 pyrosequencing*

BrdU incubation took place for ~6 h before the samples were filtered through 0.22 µm pore-size filters (Sterivex, EMD Millipore, Billerica, MA, USA) to collect bacteria. An additional size fractionation was performed with 8 µm pore-size polycarbonate filters (EMD Millipore) to include 16S rRNA sequencing analysis of particle attached bacteria in microcosm Spawn samples. Samples were immediately flash frozen in liquid nitrogen and stored frozen until processed.

Genomic DNA extraction was carried out using a ChargeSwitch Forensic DNA purification Kit (Invitrogen, Carlsbad, CA, USA). The extraction step was performed twice on the same sample to maximize DNA yield. The extracted DNAs were concentrated using Amicon Ultra-15 centrifugal filter units, 30K (EMD Millipore). The concentrated DNA solutions were purified with a NucleoSpin gDNA Clean-up Kit (Macherey-Nagel, Düren, Germany) in accordance with manufacturer's instructions.

PCR amplification was carried out in triplicate using Ex Taq HS DNA Polymerase (TaKaRa Bio., Shiga, Japan). The denaturation step was done at 94 °C for 3 minutes, followed by 25 or 30 cycles at 98 °C for 10 seconds. Primer annealing took place for 30 seconds at 55 °C. Extension took place at 72 °C for 1 minute, followed by a final extension at 72 °C for 3 minutes. The PCR products were purified with Agencourt Ampure XP Kit (Beckman Coulter, Brea, CA, USA) in accordance with the manufacturer's instructions. Equal amounts of the PCR amplicons from different samples were mixed. Pyrosequencing of the amplicon mixture was performed using a GS Junior Titanium emPCR Lib-L Kit (Roche Diagnostics, Branford, CT, USA) and a 454 GS Junior system (Roche Diagnostics).

Processing and analyses of the pyrosequencing data were performed using mothur v 1.35.1 (Scholss et al, 2009). Sequencing errors and low-quality sequences in the raw sequencing data were removed in accordance with the standard 454 operating procedure (Scholss et al, 2011). A distance matrix was constructed for the remaining sequences, and the sequences were clustered into operational taxonomic units (OTUs) at 97% similarity level with average neighbor clustering. Representative sequences within each OTU were classified with a *k*-mers nearest neighbor searching method (Wang & Qian, 2009) against SILVA 119 reference database (Yilmaz et al, 2014) at 80% threshold. Unknown sequences, as well as sequences affiliated to Eukaryotes, Chloroplast, and Mitochondria were eliminated. Read lengths ranged from 350 to 449 base pairs (bp) after quality filtering (average read length = 388 bp) and there were 10,419 ± 6825 reads sample^-1^.

The resulting OTU table was imported to R using the Phyloseq package version 1.19.1 (McMurdie and Holmes, 2013) and normalized using the cumulative-sum scaling method with the MetagenomeSeq package version 1.16.0 (Paulson et al, 2013). Normalized OTU counts were transformed to represent percentages of each sample’s total in order to perform comparisons of relative abundance. To calculate taxa consistently found in BrdU-labeled DNA from the Spawn microcosms, Phyloseq scripts were used to subset the data to only Spawn_0h_ and Spawn_66h_ samples; as well as to filter out rare OTUs of < 0.1% mean relative abundance across samples, and/or found only in a single replicate for each time point. To evaluate the contribution of taxa which were observed only in the BrdU-labled DNA and not the Total DNA, Phyloseq and base R commands were similarly used to identify those taxa unique to the BrdU-labeled samples and to determine their mean relative abundance in each subset. The MetagenomeSeq-normalized count table served as direct input to differential abundance analysis with DESeq2 version 1.14.1 (Love et al, 2013) to identify OTUs enriched in the particle-associated fraction. P-values were corrected for multiple testing using the Benjamini-Hochberg method (Benjamini & Hochberg, 1995). Output statistics of the DESeq2 analysis were filtered to include only those for taxa which were enriched or depleted in the particle fraction by log2-fold-change ≥ 2 (4-fold change), and with corrected p-values < 0.05. The results of these analyses were plotted using ggplot2 version 2.2.1 (Wickham, 2016).

References

Robinson C. (2008). Heterotrophic Bacterial Respiration. In: Kirchman, D (ed). Microbial Ecology of the Oceans. John Wiley & Sons, Inc., pp 299-334.

Del Giorgio P, Cole J. (1998). Bacterial growth efficiency in natural aquatic systems. *Annu Rev Ecol Evol Syst* **29**.

Meyer JL, Edwards JT, Risley R. (1987). Bacterial Growth on Dissolved Organic Carbon from a Blackwater River. *Microb Ecol* **13:** 13-29.

Kirchman DL, Suzuki Y, Garside C, Ducklow HW. (1991). High turnover rates of dissolved organic carbon during a spring phytoplankton bloom. *Nature* **352:** 612-614.

Smith D, Steward G, Long R, Azam F. (1995). Bacterial mediation of carbon fluxes during a diatom bloom in a mesocosm. *Deep Sea Research Part II: Topical Studies in Oceanography* **42:** 75-97.

Koch BP, Dittmar T, Witt M, Kattner G. (2007). Fundamentals of molecular formula assignment to ultrahigh resolution mass data of natural organic matter. *Anal Chem* **79:** 1758–1763.

Lechtenfeld OJ, Koch BP, Gašparovi´C B, Frka S, Witt M, Kattner G. (2013). The influence of salinity on the molecular and optical properties of surface microlayers in a karstic estuary. *Mar Chem* **150:** 25-38.

Scholss PD, Westcott SL, Ryabin T. (2009). Introducing mothur: open-source, platform-independent, community-supported software for describing and comparing microbial communities. *Appl Environ Microbiol* **75:** 7537–7541

.

Scholss PD, Gevers D, Westcott SL, . (2011). Reducing the effects of PCR amplification and sequencing artifacts on 16S rRNA-based studies. . *PLoS One* **6**.

Wang Y, Qian PY. (2009). Conservative fragments in bacterial 16S rRNA genes and primer design for 16S ribosomal DNA amplicons in metagenomic studies. . *PLoS One* **4**.

Yilmaz P, Parfrey LW, Yarza P, Gerken J, Pruesse E, Quast C*, et al.* (2014). The SILVA and ‘all-species Living Tree Project (LTP)’ taxonomic frameworks. *Nucleic Acids Res* **42:** D643–D648.

Paulson JN, Stine OC, Bravo HC, Pop M. (2013). Differential abundance analysis for microbial marker-gene surveys. *Nat Methods* **10:** 1200-1202.

Love M, Anders S, Huber W, Love MM, Biocgenerics I, Biobase B*, et al.* (2013). Package ‘DESeq2’.

Benjamini Y, Hochberg Y. (1995). Controlling the false discovery rate: a practical and powerful approach to multiple testing. *J R Stat Soc Ser B* **57:** 289–300.

Wickham H. (2016). ggplot2: elegant graphics for data analysis. *Springer. Vancuver*.
