# Supplementary figures and images for: Bacterioplankton drawdown of coral mass-spawned organic matter
Source: ISME J. 2018 Jun 8;12(9):2238–51. doi: 10.1038/s41396-018-0197-7 (PMC6092384; doi:10.1038/s41396-018-0197-7)

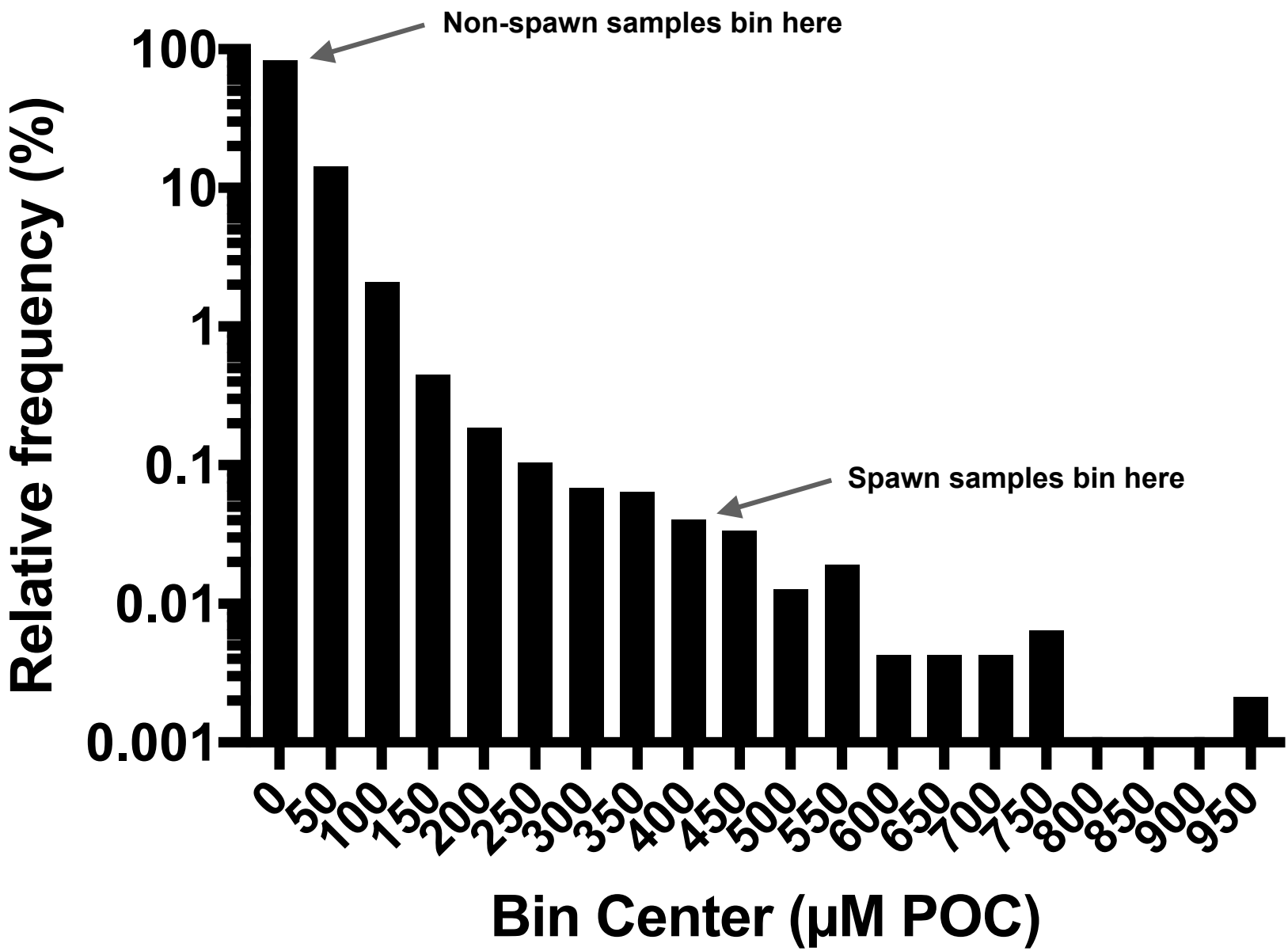

Supplement: Supplementary file 2 — Figure S1 [file 41396_2018_197_MOESM2_ESM.pdf]

0 h

a

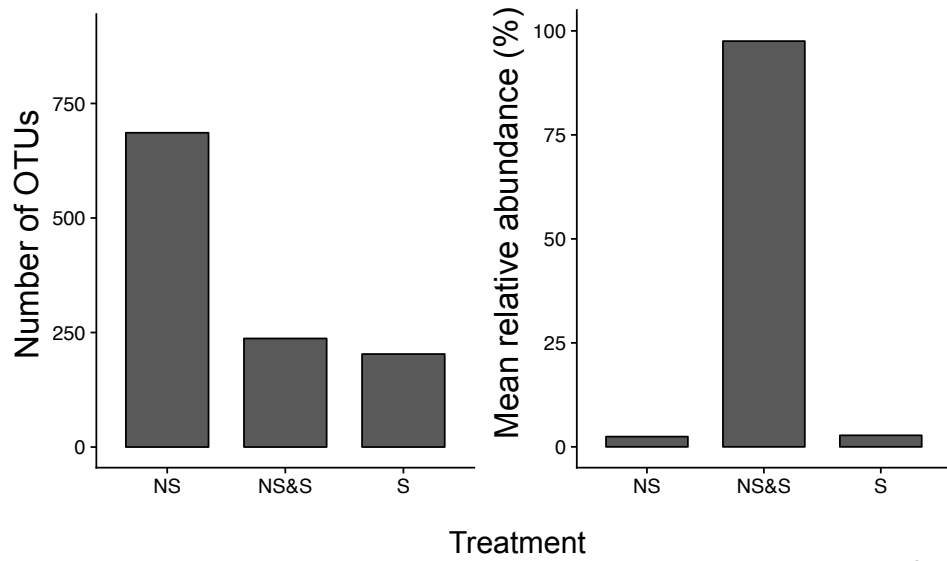

66 h

b

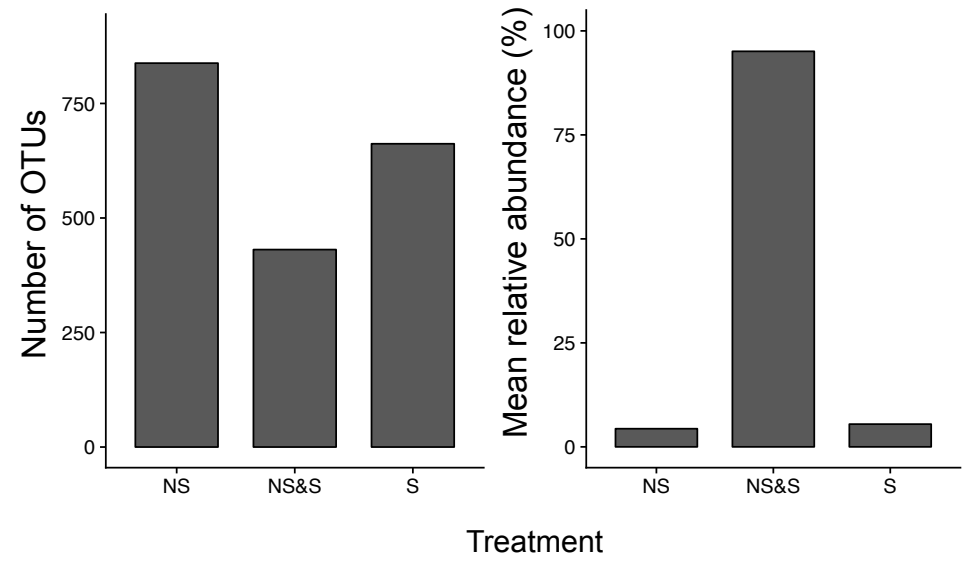

NS = NonSpawn  
S = Spawn  
NS&S = NonSpawn & Spawn

Supplement: Supplementary file 3 — Figure S2 [file 41396_2018_197_MOESM3_ESM.pdf]

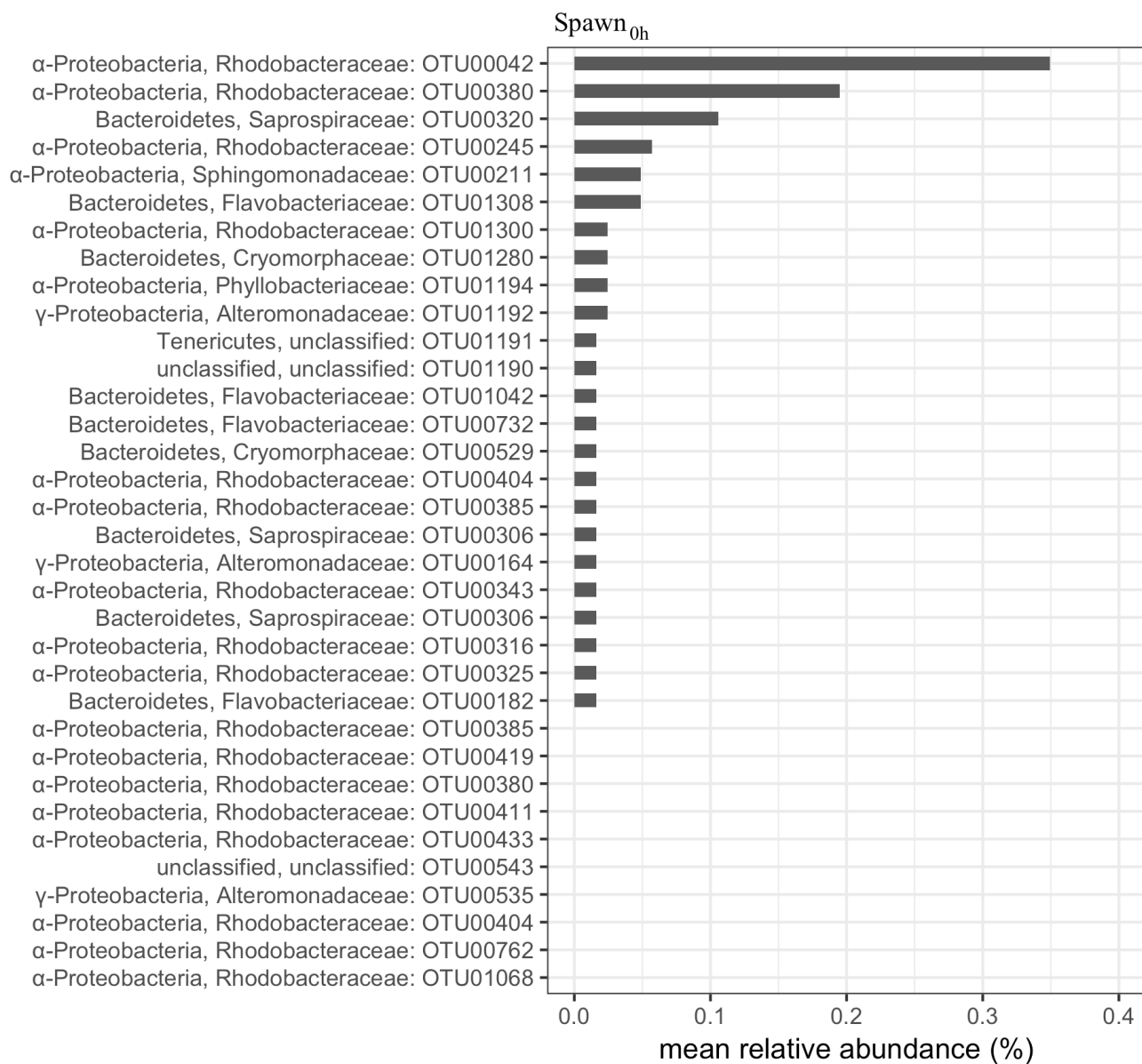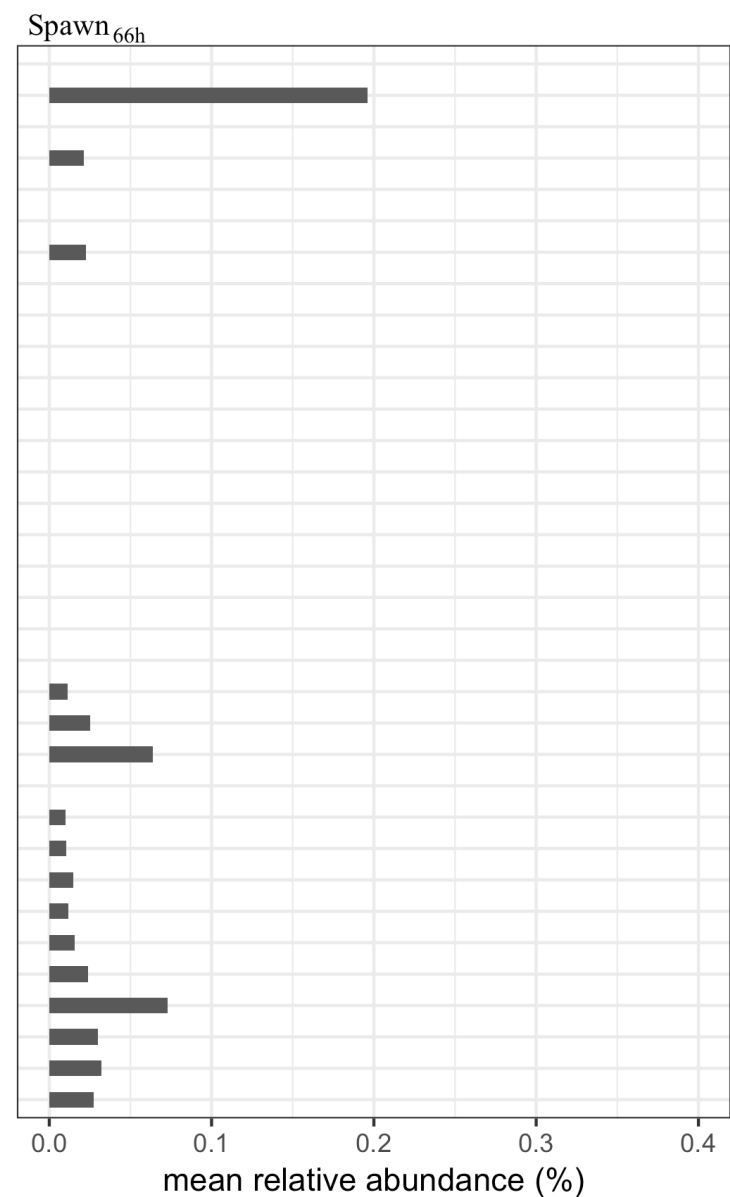

Supplement: Supplementary file 4 — Figure S3 [file 41396_2018_197_MOESM4_ESM.pdf]

OTUs with Phylum, Family association

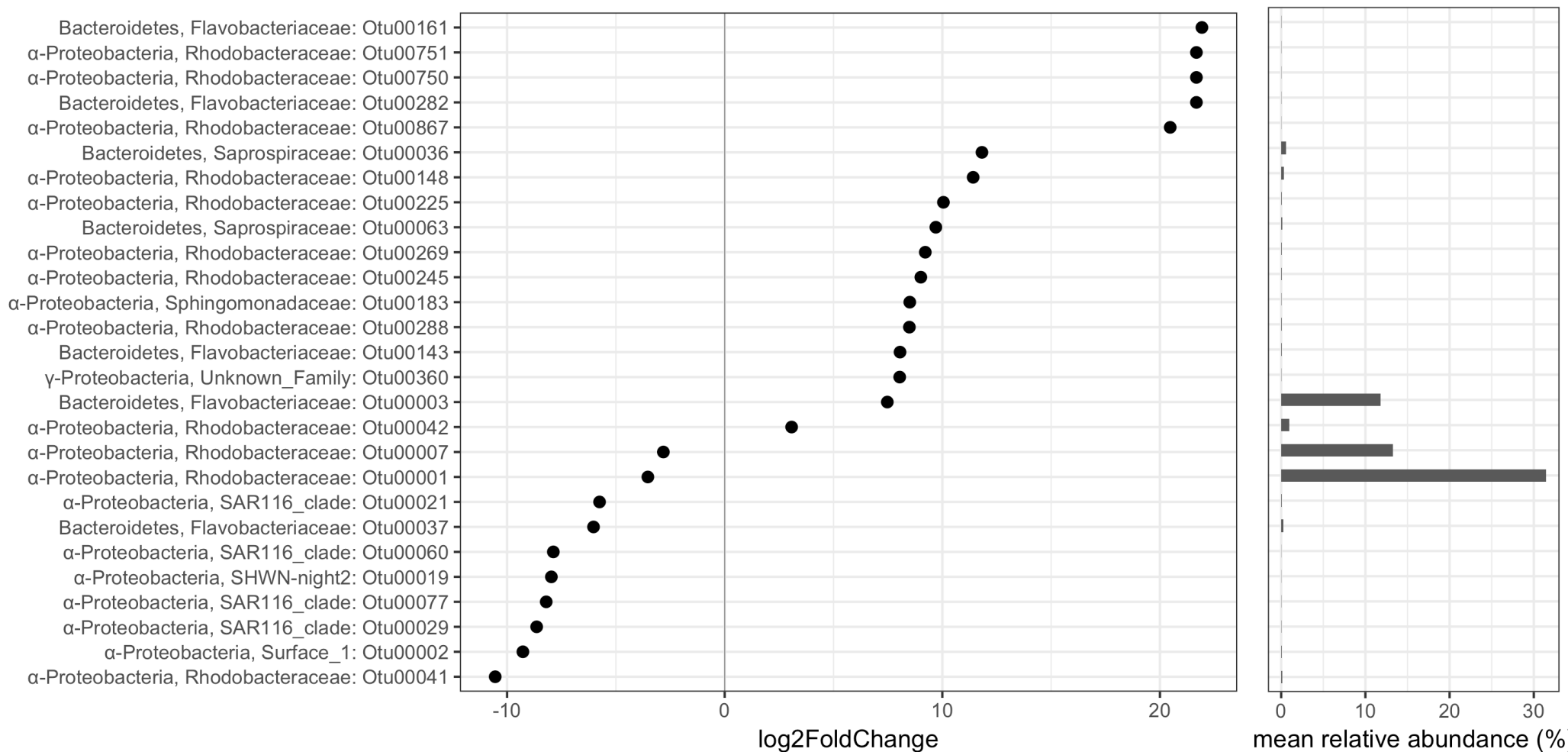

Supplement: Supplementary file 5 — Figure S4 [file 41396_2018_197_MOESM5_ESM.pdf]
