# Supplementary material for: Bacterioplankton drawdown of coral mass-spawned organic matter
Source: ISME J. 2018 Jun 8;12(9):2238–51. doi: 10.1038/s41396-018-0197-7 (PMC6092384; doi:10.1038/s41396-018-0197-7)
Supplement: Supplementary file 10 — Supplementary Figure Legends [file 41396_2018_197_MOESM10_ESM.docx]

**Supplementary figure legends**

Figure S1. Compiled POC measurements from 46 937 global samples (Martiny et al, 2014): range, 0–927.1; median, 7.4; 25% percentile, 2.8; 75% percentile, 18.1. Arrows: Non-spawn POC measurements fell within the < 25 µM POC bin, with 82.5% of the samples; Spawn POC measurements fell into the 375–425 µM POC bin. Only ~0.1% of the samples (n = 49) from the compiled data had higher POC concentration than microcosm Spawn_0h_ samples. y-axis, relative frequency (%) of samples contained in bins; x-axis, histogram bin increments of 50 µM POC.

Figure S2. Number of taxa and summed mean relative abundances of OTUs from the Total DNA in 0 h samples (a) and 66 h samples (b). S, Spawn; NS, Non-spawn; NS & S, Non-spawn and Spawn.

Figure S3. Mean relative abundance (%) of active-but-rare bacterial taxa found in microcosm Spawn. Shown taxa were present in at least one biological replicate and comprised ≥ 0.01% relative abundance in the sample. Class designations: α, Alphaproteobacteria; $\gamma$, Gammaproteobacteria.

Figure S4. Log2-fold change (left) and mean relative abundance (%) (right) for the BrdU-labeled taxa associated with > 8 µm particles from microcosm Spawn 0 h to 66 h. Class designations: α, Alphaproteobacteria; $\gamma$, Gammaproteobacteria.
